# Optimized 5-Fluorouridine Prodrug for Co-Loading with Doxorubicin in Clinically Relevant Liposomes

**DOI:** 10.3390/pharmaceutics13010107

**Published:** 2021-01-15

**Authors:** Debra Wu, Douglas Vogus, Vinu Krishnan, Marta Broto, Anusha Pusuluri, Zongmin Zhao, Neha Kapate, Samir Mitragotri

**Affiliations:** 1 John A. Paulson School of Engineering and Applied Sciences, Harvard University, Cambridge, MA 02138, USA; debrawu@g.harvard.edu (D.W.); dvogus37@gmail.com (D.V.); vinu@g.harvard.edu (V.K.); anusha@g.harvard.edu (A.P.); zmzhao@g.harvard.edu (Z.Z.); nkapate@mit.edu (N.K.); 2 Wyss Institute of Biologically Inspired Engineering, Harvard University, Boston, MA 02115, USA; 3 Department of Materials, Department of Bioengineering and Institute of Biomedical Engineering, Imperial College London, South Kensington, London SW7 2BU, UK; m.broto-aviles@imperial.ac.uk; 4 Nanobiotechnology for Diagnostics (Nb4D), Institute for Advanced Chemistry of Catalonia of the Spanish Council for Scientific Research (IQAC-CSIC), 08034 Barcelona, Spain; 5 Harvard-MIT Division of Health Sciences and Technology, Massachusetts Institute of Technology, Cambridge, MA 02139, USA

**Keywords:** liposome, drug combination, nanoparticle, targeting

## Abstract

Liposome-based drug delivery systems have allowed for better drug tolerability and longer circulation times but are often optimized for a single agent due to the inherent difficulty of co-encapsulating two drugs with differing chemical profiles. Here, we design and test a prodrug based on a ribosylated nucleoside form of 5-fluorouracil, 5-fluorouridine (5FUR), with the final purpose of co-encapsulation with doxorubicin (DOX) in liposomes. To improve the loading of 5FUR, we developed two 5FUR prodrugs that involved the conjugation of either one or three moieties of tryptophan (W) known respectively as, 5FUR−W and 5FUR−W_3_. 5FUR−W demonstrated greater chemical stability than 5FUR−W3 and allowed for improved loading with fewer possible byproducts from tryptophan hydrolysis. Varied drug ratios of 5FUR−W: DOX were encapsulated for in vivo testing in the highly aggressive 4T1 murine breast cancer model. A liposomal molar ratio of 2.5 5FUR−W: DOX achieved a 62.6% reduction in tumor size compared to the untreated control group and a 33% reduction compared to clinical doxorubicin liposomes in a proof-of-concept study to demonstrate the viability of the co-encapsulated liposomes. We believe that the new prodrug 5FUR−W demonstrates a prodrug design with clinical translatability by reducing the number of byproducts produced by the hydrolysis of tryptophan, while also allowing for loading flexibility.

## 1. Introduction

Innovation in nanomedicine has resulted in the successful development of many novel oncology therapies in the clinic [1,2]. Advancements in the formulation of new nanomedicines have coincided with the identification of new therapeutic targets [3], a new understanding of the immunogenic effects of nanocarriers [4], and advancements in the delivery of biologics [5]. Still, a large majority of clinically approved nanocarriers deliver small molecule chemotherapeutics. Chemotherapeutics remain the front-line treatment for many cancers [6,7,8], and while cancer treatment has evolved with the introduction of several experimental and improved immunotherapies, chemotherapy continues to remain one of the main pillars of cancer therapy. However, intravenous administration of chemotherapy is non-specific and requires large doses to be effective, which often causes patient myelosuppression [6]. To mitigate the toxic effects of large doses, nanoparticle-based delivery systems for chemotherapeutics were developed to protect the drug cargo from enzymatic degradation and extend drug circulation time, allowing for enhanced efficacy at lower doses. Early clinically approved drug delivery systems included liposomal doxorubicin (Doxil^®^) and nanoparticle albumin-bound paclitaxel (Abraxane^®^) [9].

However, unlike most clinical chemotherapy regimens which use drug combinations to reduce the development of tumor drug resistance [10], a majority of clinically approved nanoparticle-based drug delivery systems are single-drug carriers. Multiple clinical trials have tested a combination of a single-drug carrier in combination with a free drug, but this method cannot control competing clearance profiles and tissue distribution, and often large doses of the free drug are used [11,12,13]. The development of dual-loaded nanocarriers can significantly enhance the efficacy of combination chemotherapy through sustained release and maintenance of a consistent drug ratio in circulation, and by improving the safety profile of both drugs.

While many such dual-drug nanocarriers exist in the drug delivery literature [14,15,16], the lack of many clinically translated dual-drug carriers demonstrates the complexity of these formulations. Differing pKa profiles, water solubilities, and oxidation and reduction potentials of drugs can make them incompatible for dual loading. To this end, prodrugs have been synthesized to allow active drug loading into nanoparticles [17,18,19]. However, prodrugs face the additional challenge of obtaining approval by a regulatory agency before the clinical translation of the formulation can begin. Any degradation byproducts must also be approved by a regulatory agency. Despite the limitations, prodrug chemistries can provide enhanced safety profiles and alter the physical properties of drugs to increase encapsulation in nanoparticles [19,20].

We have previously reported a polyethylene glycol-containing (PEGylated) liposomal formulation comprised of 5-fluorouridine (5FUR) and doxorubicin (DOX) for breast cancer therapy, a formulation referred to as DAFODIL (Doxorubicin and 5-fluouridine Optimally Delivered In Liposomes) [21]. 5-fluorouridine is a ribosylated nucleoside form of 5-fluorouracil, an antimetabolite drug commonly used with doxorubicin in the treatment of breast cancer [22,23]. In the previous work, a prodrug of 5FUR with three moieties of tryptophan attached via ester bonds (5FUR−W_3_) was synthesized to improve liposomal loading. However, uncontrolled hydrolysis of the three moieties of tryptophan led to the formation of a variety of possible species within the liposome, which caused significant characterization and translational challenges. In our current work, we sought to control the hydrolysis through the development of a different prodrug with only one moiety of tryptophan conjugated by ester bond to the primary alcohol of 5FUR (5FUR−W). The monoester derivative 5FUR−W was found to be more stable than the triester 5FUR−W_3_ and hydrolyzed only to the starting material 5FUR. We synthesized liposomal formulations comprising of a range of 5FUR−W: DOX ratios. Finally, we compared DAFODIL variations against a clinically bioequivalent generic formulation of Doxil known as Doxorubicin Hydrochloride Liposome Injection (DLI). Compared to DLI, DAFODIL exhibited enhanced therapeutic efficacy with no off-target toxicity in preclinical testing using the 4T1 orthotopic murine breast cancer model. We believe the optimized DAFODIL formulation represents a potential translational alternative to clinically used Doxil.

## 2. Materials and Methods

### 2.1. Liposome and Cell Culture Materials

The lipids 1,2-distearoyl-sn-glycero-3-phosphocholine (DSPC) and 1,2-distearoyl-sn-glycero-3-phosphoethanolamine-*N*-[methoxy(polyethylene glycol (PEG))-2000] (DSPE-mPEG2000) were purchased from Avanti Polar Lipids (Alabaster, AL, USA). Cholesterol was purchased from Millipore Sigma (Burlington, MA, USA). Doxorubicin (DOX) was purchased from LC labs (Woburn, MA, USA). 5-fluorouridine with a tryptophan conjugated by ester bond linkage on the primary alcohol (5FUR−W) was a prodrug based on 5-fluorouracil synthesized by Pharmaron (Beijing, China). 5-flurouridine with tryptophan conjugated to each alcohol (5FUR−W_3_) was a prodrug synthesized by Dr. Reddy’s Laboratories. Doxorubicin Hydrochloride Liposome Injection (DLI) was also provided by Dr. Reddy’s Laboratories (Hyderabad, India).

4T1 murine breast cancer cells (ATCC CRL-2539) were purchased from ATCC (Manassas, VA, USA). 4T1 cells were grown in RPMI-1640 cell growth media supplemented with 10% fetal bovine serum (FBS) and 1% penicillin/streptomycin. SKOV-3 cells were grown in McCoy’s 5a Medium Modified supplemented with 10% fetal bovine serum (FBS) and 1% penicillin/streptomycin. Cellular inhibition assays used 3-(4,5-dimethylthiazol-2-yl)-2,5-diphenyltetrazolium bromide (MTT) to quantify cell viability. All aforementioned cell culture materials were purchased from ThermoFisher Scientific (Waltham, MA, USA). Cell culture flasks and tissue culture-treated well plates were purchased from Corning (Corning, NY, USA).

### 2.2. Liposome Fabrication

Liposomes were fabricated through the conventional thin film hydration method [24]. 40 µmol of lipids (56.4% DSPC, 5.3% DSPE-mPEG2000, 38.3% cholesterol) were dissolved in 300 µL of chloroform and dried under vacuum using rotary evaporation and heating at 65 °C. The lipid film was resuspended in 1.1 mL of ammonium sulfate buffer (250 mM, pH 5.5). The solution was sonicated and extruded through a 50 nm polycarbonate membrane purchased from Avestin (Ottawa, ON, Canada) to form unilamelar liposomes. Approximately 1 mL of liposomes were then passed through a PD-10 size exclusion column purchased from GE Healthcare (Piscataway, NJ, USA) that had been equilibrated with PBS prior to use. Removal of extraliposomal ammonium sulfate salts by size exclusion created a pH gradient to facilitate the loading of drugs. Doxorubicin was then incubated with the liposomes for 2.5 h at 65 °C, followed by 5FURW for an additional hour. For a liposomal ratio of 2.5 5FURW: DOX, 100 µL of 175 mg/mL 5FURW and 50 µL of 40 mg/mL DOX was used. For a liposomal ratio of 6 5FURW: DOX, 5FURW was adjusted to 100 µL of 200 mg/mL and DOX was decreased to 50 µL of 20 mg/mL. Free drug was removed using size exclusion chromatography with PBS as the mobile phase.

### 2.3. Liposome Characterization

Liposomal size and zeta potential were quantified by measurement of the number size distribution and zeta potential distribution by a Malvern Zetasizer. Liposomes were diluted 100-fold in milliQ water prior to analysis. Liposomal drug content was quantified by reverse phase HPLC. An example of the free drug HPLC chromatogram is given in Figure S1. Liposomes were disrupted by 10-fold dilution in 1:1 methanol: acetonitrile with 0.05% formic acid. The solution was then sonicated for 30 min and centrifuged. The supernatant was removed and further 10-fold diluted in water with 0.1% formic acid. 10 µL of the sample was injected into a Waters HPLC system fitted with a Zorbx 300Extend C18 3.5 μm column (150 × 4.6 mm) purchased from Agilent (Santa Clara, CA, USA). The column was equilibrated with 0.5 mL/min 99% mobile phase A (water with 0.1% trifluoroacetic acid) and 1% mobile phase B (acetonitrile with 0.1% trifluoracetic acid) at the time of sample injection. The gradient gradually changed to 60% mobile phase B at 10 min.

### 2.4. Prodrug Hydrolysis and Storage Stability Study

1 mM solutions (*n* = 3) of both monoester 5FUR−W and triester 5FUR−W_3_ were prepared in milliQ water and kept at 65 °C with stirring. At each timepoint (0 min, 10 min, 30 min, 1 h, 2 h, 3 h, and 4 h) a 10 µl sample was taken from the stock solutions and diluted 100-fold in milliQ water. The samples were run on HPLC using the previously described method for drug quantification.

To quantify the storage stability of DAFODIL, DAFODIL_R=2.5_ (*n* = 3) was stored in PBS at 25 °C for five days, and at 4 °C for six days. A 10 µL sample was taken each day from the DAFODIL_R=2.5_ staored at 25 °C, and on day 0, 1, 2, 4, and 6 from the DAFODIL_R=2.5_ stored at 4 °C. The samples were run on HPLC using the previously described method for drug quantification.

### 2.5. Cellular Inhibition Assay

4T1 cells were plated in 96-well plates at a seeding density of 500 cells/well, and SKOV-3 cells were plated at a density of 5000 cells/well. Cell culture media containing treatment were administered onto six wells per treatment. Treatment was incubated on the cells for 72 h before the removal of the cell media and administration of 0.5 mg/mL MTT reagent in media. After 5 h, formazan crystals were dissolved in DMSO and absorbance was measured by plate reader.

### 2.6. Tumor Model

Murine breast cancer tumors were established by subcutaneous injection of 10^5^ 4T1 cells in 50 µL of PBS into the fourth abdominal mammary fat pad of female BALB/c mice (age 50–56 days) purchased from Charles River Laboratories (Wilmington, MA, USA). This method has proven to produce uniform breast cancer tumors that grow rapidly and metastasize to the lungs [25,26]. Tumors volume was calculated with $V=\left(\frac{1}{2}\right)L\times W^{2}$, where *L* and *W* represent the length and width of the tumor, as determined through caliper measurements. Treatment with liposomal formulations was initiated when tumors reached 50 mm^3^ in size. Treatment consisted of 100 µL of liposomes administered intravenously every other day a total of four times. Liposomes were adjusted to a dose of 3 mg/kg DOX, and the accompanying 5FUR−W dosage was determined by the molar ratio of 5FUR−W: DOX encapsulated in liposomes (Table S1). Each group was injected a total of four times with a day in between injections to prevent toxic buildup of the long-circulating liposomes.

### 2.7. Pharmacokinetics and Biodistribution

Liposomal pharmacokinetics were studied by intravenous injection of liposomes (100 µL, 1.5 mg/kg DOX) in healthy female BALB/c mice. Blood was collected by mandibular puncture at 5 min (~20 μL) and by cardiac puncture at 2, 6, and 24 h (~200 μL) after injection and stored in heparin-coated collection tubes from BD (Franklin Lakes, NJ, USA). Blood collected by mandibular puncture was diluted five-fold with PBS and blood collected by cardiac puncture was diluted two-fold in PBS. 100 μL of the blood and PBS mixture was centrifuged at 7000 g for 10 min to remove the red blood cells. The supernatant was then 10-fold diluted in 1:1 methanol: acetonitrile with 0.05% formic acid for drug extraction. The sample was centrifuged to remove serum proteins and the resulting supernatant was filtered with 0.2 μm syringe filters from Waters (Milford, MA, USA) and run using the drug quantification protocol on LC-MS. The mass spectrometer was used to determine if the metabolite forms of the drugs were present in the plasma. Finally, pharmacokinetic parameters for the one-compartment model were calculated using the PK Solver 2.0 Microsoft Excel plugin developed by Zhang et al. [27].

Liposomal biodistribution was performed by intravenous injection of liposomes at a dose of 1.5 mg/kg DOX into BALB/c mice. Mice were euthanized 24 h after injection, and the tumor, liver, lungs, and kidneys were extracted and weighed. The samples were cut into small pieces and enzymatically digested for one hour at 37 °C in 5 mg/mL of collagenase and 50 U/mL of DNAse in HBSS buffer. Samples were further broken down with a homogenizer probe and centrifuged at 20,000 g for 5 min. Drug content was extracted from the samples by 5-fold dilution in 1:1 methanol: acetonitrile with 0.1% formic acid. The resulting mixture was sonicated for 15 min and centrifuged to remove the homogenized material. The supernatant was filtered using 0.2 µm syringe filters from Waters (Milford, MA, USA) and analyzed using the aforementioned reversed phase HPLC method.

### 2.8. Statistical Analysis

Statistical significance was determined through unpaired t-tests and one-way ANOVA with Tukey’s Multiple Comparison Test in Graphpad v5 (produced by Graphpad Software, San Diego, CA, USA, 2008). Statistical significance was defined as * *p* < 0.05, ** *p* < 0.01, and *** *p* < 0.001.

## 3. Results

### 3.1. Comparison of 5FUR Prodrug Stability and In Vitro Toxicity

**Figure 1:**
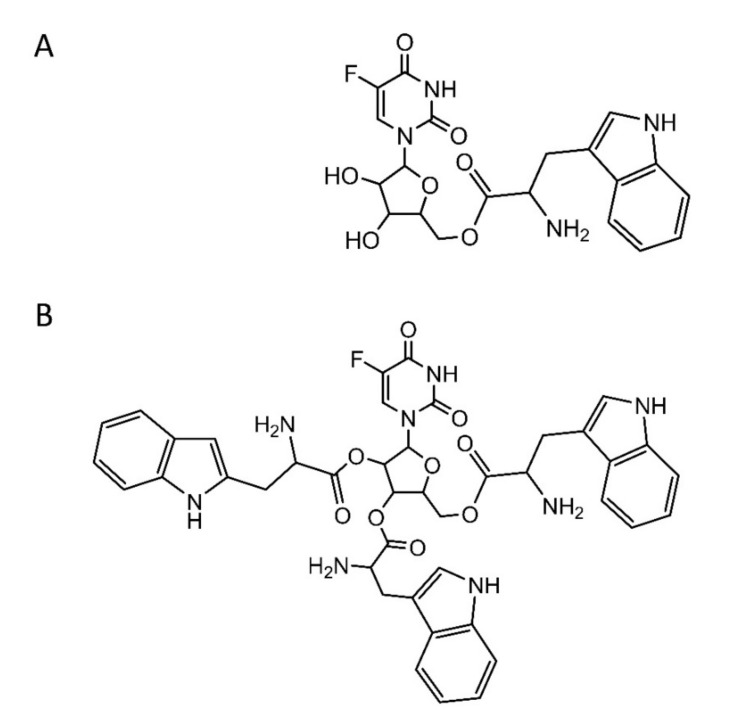
Prodrugs used were designed with the conjugation of tryptophan (W) to 5-fluorouridine to make the compound weakly basic and favorable to ammonium sulfate gradient loading in liposomes. (**A**) The monoester 5FUR−W has tryptophan conjugation to the primary alcohol of 5FUR. (**B**) The triester 5FUR−W_3_ has tryptophan conjugated to every alcohol of 5FUR.

**Figure 2:**
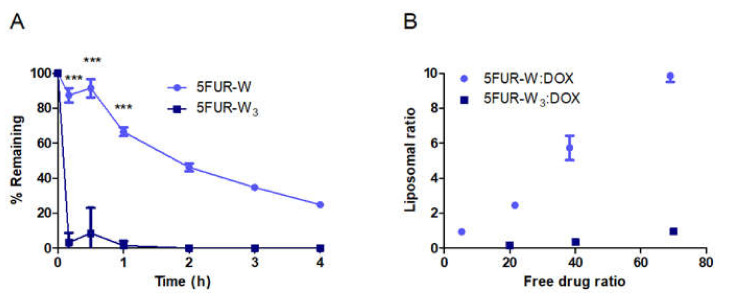
5FUR−W was determined to be the more stable prodrug for proceeding with liposomal loading. (**A**) Hydrolysis of 5FUR−W and 5FUR−W_3_ showed that 5FUR−W is significantly more stable in heated solution (*p* < 0.01). (**B**) 5FUR−W achieved a large range of liposomal ratios when loaded in tandem with DOX. 5FUR−W_3_ was not as efficient in loading.

In previous work, 5FUR−W_3_ facilitated active loading into liposomes. However, multiple hydrolysis products complicated the characterization of the formulation, and 5FUR−W was developed to provide better stability and higher loading. The thermal stability of the 5FUR−W monoester (Figure 1A) was compared to the triester 5FUR−W_3_ (Figure 1B) at 65 °C, the temperature at which drug loading occurs. Less than 10% of 5FUR−W_3_ was detected after 10 min, showing that at least one of the three tryptophan ester bonds was highly labile. 5FUR−W_3_ had completely converted to other forms after 1 h. This implies that the triester prodrug rapidly hydrolyzes to dual-ester and monoester forms upon heating in solution, which makes it nonideal for liposomal loading. However, approximately 66% of 5FUR−W was conserved after 1 h of heating, making it significantly more stable than 5FUR−W_3_ (Figure 2A). Furthermore, 5FUR−W can only hydrolyze to free tryptophan and 5FUR.

Liposomes possessing a wide range of 5FUR−W: DOX ratios from 1 to 10 were produced using different free drug ratios of 5FUR−W: DOX during liposomal loading (Figure 2B). In contrast, 5FUR−W_3_ resulted in ratios less than 1:1 5FUR−W_3_: DOX in the final liposomal sample. 5FUR−W was therefore used in the fabrication of DAFODIL. Regardless of 5FUR−W:DOX ratio, size and zeta potential of DAFODIL remained comparable to that of single DOX-loaded liposomes (DOX-L) (Table 1). Liposomal size and zeta potential were similar to that of other PEGylated liposomal formulations reported in the literature [4,28,29]. A slightly negative zeta potential has previously been linked to reduced liver uptake of nanoparticles and prolonged nanoparticle circulation time [30].

**Figure 3:**
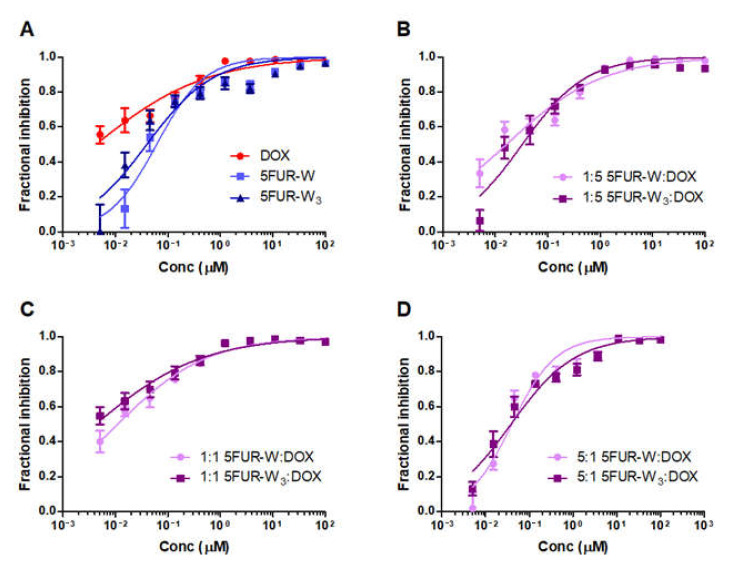
In vitro comparison of 5FUR−W and 5FUR−W_3_ dose-response cellular fractional inhibition on 4T1 breast cancer cells showed a high degree of similarity. All points represent *n* = 6. (**A**) Comparison of free single drugs DOX, 5FUR−W, and 5FUR−W_3_. (**B**) Comparison of 1:5 ratio of 5FUR prodrugs to DOX. (**C**) Comparison of 1:1 ratio of 5FUR prodrugs to DOX. (**D**) Comparison of 5:1 ratio of 5FUR prodrugs to DOX.

The toxicity of 5FUR−W and 5FUR−W_3_ were compared in vitro on the 4T1 murine breast cancer cell line. Free drugs and free drug combinations of 1:5, 1:1, and 5:1 of prodrug to DOX were evaluated. Free 5FUR−W had a similar dose-response to 5FUR−W_3_, although free DOX had approximately 10-fold lower IC_50_ than both prodrugs (Figure 3A). The IC_50_ values of 5FUR−W and 5FUR−W_3_ were all similar across the comparison groups (Figure 3B–D). Dose response parameters are given in Table S2. Limited drug synergy was observed between doxorubicin and the 5-fluorouridine prodrugs on the 4T1 cell line.

Treatments consisting of DOX-L, 5FUR−W-L, and a 2.5 molar ratio 5FUR−W-L to DOX-L were compared to DAFODIL_R=2.5_ in vitro on the 4T1 and SKOV3 cell lines. 5FUR−W-L exhibited a lower IC_50_ than DOX-L in each cell line (Figure S2), which may be due to accelerated release kinetics of 5FUR−W-L compared to the very stably loaded DOX-L. However, the IC_50_ of DAFODIL_R=2.5_ was fairly similar to molar ratio 2.5 5FUR−W-L + DOX-L in 4T1 cells. In the SKOV-3 cell line, the IC_50_ of DAFODIL_R=2.5_ was three-fold lower than the combined liposome group. This may indicate potential efficacy in the SKOV-3 tumor model. Dose–response parameters are given in Table S3.

### 3.2. DAFODIL Conserved Drug Ratio in Circulation

**Figure 4:**
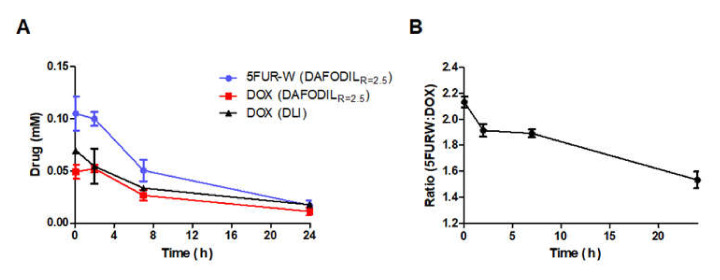
Drug pharmacokinetics of liposomal formulations showed sustained release. All formulations were injected inTable 1. 5 mg/kg DOX (*n* = 3) (**A**) Drug plasma concentrations of 5FUR−W and DOX from DAFODIL_R=2.5_ and DOX from DLI. (**B**) DAFODIL_R=2.5_ drug plasma ratio over time.

A formulation of DAFODIL_R=2.5_ was compared to Doxorubicin hydrochloride Liposome Injection (DLI), a clinically equivalent generic form of Doxil (AB-rated). All formulations were injected in equal doses and observed over a 24 h period. The gradual decrease in DOX concentration of DLI and DAFODIL was similar, with half-lives of 14.79 ± 4.11 and 10.47 ± 2.02 h respectively (Figure 4A). The 5FUR−W concentration in DAFODIL had a half-life of 8.81 ± 1.43 h. Due to the similarity in pharmacokinetic profile, the drug ratio of 5FUR−W: DOX was 71.4% conserved at the end of 24 h (Figure 4B). Furthermore, the biodistribution of doxorubicin in the tumor was similar between all tested groups (Figure S3). All pharmacokinetic parameters can be found in Table 2.

### 3.3. DAFODIL Demonstrates Superior Tumor Efficacy to DLI

Given the similarity of pharmacokinetic profile between DAFODIL and DLI and the maintenance of the 5FUR−W:DOX ratio in circulation, the formulations were compared in the 4T1 orthotopic murine breast cancer model in female BALB/c mice. This tumor model has been shown to exhibit rapid and aggressive growth, with metastasis to the lungs representative of human breast cancer [26,31]. DOX and 5-fluoropyrimidine analogs, the basis of the 5FURW prodrug, are commonly used as part of breast cancer treatment regimens [32,33]. A range of ratios of 5FUR−W: DOX encapsulated in DAFODIL were evaluated in the 4T1 tumor model and compared to the clinically available DLI formulation. All dosages had an equivalent DOX concentration of 1 mg/kg. This in vivo study delivered proof-of-concept confirmation that the addition of an optimized liposomal prodrug can boost the performance of doxorubicin liposomes after adjusting dosage.

**Figure 5:**
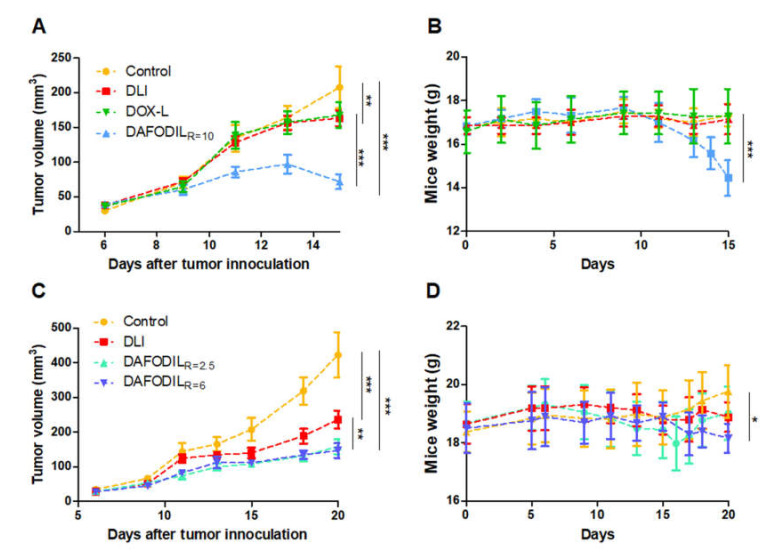
DAFODIL was compared to DLI in the 4T1 tumor model using different 5FURW:DOX ratios. (**A**) DAFODIL_R=10_ significantly reduced tumor growth. All formulations began dosing on day 6, with four administrations spaced every two days. (**B**) DAFODIL_R=10_ caused significant weight loss in treated mice. (**C**) All formulations were administered on day 6. DAFODIL_R=2.5_ (four injections, every two days, *n* = 6) was similar to DAFODIL_R=6_ (4 injections, every 4 days, *n* = 5) and both significantly reduced tumor volume compared to DLI (four injections, every two days, *n* = 6) (**D**) While DAFODIL_R=6_ caused significantly different weight loss, DAFODIL_R=2.5_ expressed no toxicity associated with weight loss. * *p* < 0.05, ** *p* < 0.01, and *** *p* < 0.001.

Initially, a high ratio of 10 5FUR−W: DOX in DAFODIL_R=10_ was compared against DLI. We also prepared single-drug doxorubicin-loaded liposomes (DOX-L) made using the same protocol as DAFODIL. DOX-L were also compared against DLI to determine if differences in fabrication procedure contribute to any significant differences in efficacy. No significant difference between DOX-L and DLI was found. DAFODIL_R=10_ exhibited exceedingly effective tumor volume control compared to both DOX-L and DLI (Figure 5A). However, this was accompanied by a decrease of over 15% of mice body weight in the DAFODIL_R=10_ group, thus necessitating the end of the study (Figure 5B). Toxicity was attributed to the high loading of 5FURW.

Liposomal ratio was lowered to R = 2.5 and R = 6 to optimize liposomal efficacy without toxicity. The dosing schedule of the four total injections of DAFODIL_R=2.5_ and DAFODIL_R=6_ was also assigned every two day and every four days, respectively. DAFODIL_R=2.5_ and DAFODIL_R=6_ had comparable efficacy, and both were significantly more effective than DLI (*p* < 0.01) (Figure 5C). No group caused the average mice weight loss to exceed 15%, but the weight of the DAFODIL_R=6_ group was significantly different (*p* < 0.05) from the control group at 20 days (Figure 5D). DAFODIL_R=2.5_ remained statistically different from DLI at 27 days (*p* < 0.001) (Figure S4).

### 3.4. Storage Stability of DAFODIL_R=2.5_ Formulation

**Figure 6:**
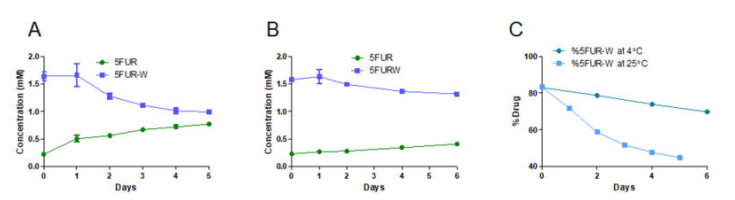
Storage stability of 5FUR−W in DAFODIL_R=2.5_ (*n* = 3). (**A**) Hydrolysis of 5FUR−W at 25 °C. (**B**) Hydrolysis of 5FUR−W at 4 °C. (**C**) Ratio of 5FUR−W to all 5FU containing species at both 4 °C and 25 °C.

We have confirmed 5FUR−W remains stable longer than 5FUR−W_3_ at the encapsulation temperature, 65 °C. Through in vivo optimization, we have concluded DAFODIL_R=2.5_ improved efficacy while also remaining nontoxic. Next, we evaluated the storage stability of 5FUR−W in DAFODIL_R=2.5_. At 25 °C, approximately 40% of initial 5FUR−W was hydrolyzed to 5FUR by the end of five days (from 1.64 mM to 0.99 mM 5FUR−W) (Figure 6A). However, when stored at 4 °C, 5FUR−W displayed slow hydrolyzation that resulted in the conversion of 17% of 5FUR−W to 5FUR (Figure 6B). Overall, the ratio of 5FUR−W hydrolysis was much quicker at 25 °C than 4 °C, but storage at 4 °C was ineffective at stopping hydrolysis completely (Figure 6C). This indicates that storage at 4 °C will likely suffice for short periods of time, but further optimization of DAFODIL should include lyoprotectants for lyophilized storage [34].

## 4. Discussion

Liposomal doxorubicin, better known as Doxil, was among the first clinically approved nanocarriers for cancer therapy [35]. With the approval of other liposomal drug formulations (DaunoXome^®^, Marquibo^®^, Onivyde^®^) and albumin nanoparticle-bound paclitaxel (Abraxane) nanomedicine has emphasized the efficacy of long-circulating formulations with sustained release [9]. These formulations have since been tested in several clinical trials [36,37,38], but the most frequently observed benefit of these formulations is reduced toxicity and not enhanced tumor efficacy [39,40,41,42]. However, the recently approved Vyxeos, a co-encapsulated daunorubicin and cytarabine liposome, has demonstrated enhanced response rate and survival time in patients with acute myeloid leukemia, thus highlighting the impact of dual-drug loaded carriers [43].

Our approach to improving liposomal doxorubicin was to incorporate a fluoropyrimidine analog, 5-fluorouridine, which metabolizes to the same active compounds as 5-fluorouracil (5FU), a drug used with doxorubicin and cyclophosphamide in the treatment of breast cancer [44,45]. We developed a 5FUR prodrug by conjugating tryptophan to the primary alcohol, resulting in the most stable ester [46]. Furthermore, 5FUR−W hydrolyzed only to 5FUR and did not present any other prodrug forms that would present a regulatory challenge. We demonstrated that by reducing prodrug hydrolysis during the drug loading process, we were capable of actively loading significantly more 5FUR−W in liposomes.

While synergy was not present in the drug combination of 5FUR−W and DOX on either the triple-negative 4T1 murine breast cancer cell line or the SKOV-3 human ovarian cancer cell line, previous work has reported synergy of the DAFODIL formulation on the BT-474 cell line, which belongs to the Luminal B subtype of breast cancer [21,47]. However, our focus was rather to improve the chemical stability of the 5FUR prodrug by changing its design. We continued in vivo testing of DAFODIL using the 4T1 orthotopic breast cancer model, due to its aggressive growth and similarity in metastasis to human breast cancer [26]. We recommend further in vitro synergy quantification on patient-derived xenografts, which would provide more clinically relevant results [48,49].

In typical clinical combination regimens, 5FU is given in large excess of DOX due to the respective clearance profile and mechanism of each drug [22,23]. While R > 1 was previously unobtainable with 5FUR−W_3_, the new single ester 5FUR−W enables the formulation of combination liposomes with R > 1. Our resulting formulations, DAFODIL_R=10_, DAFODIL_R=6_, and DAFODIL_R=2.5_, were compared against an AB-rated generic liposomal doxorubicin formulation, DLI. As a commercially used drug, DLI was the most stringent control formulation to represent liposomal doxorubicin. 5FUR−W-L was not included as a control due to the lack of a clinical equivalent. The purpose of this preliminary animal study was to evaluate if DAFODIL could leverage the incorporation of the 5FUR−W prodrug to achieve improved tumor control over the closest clinical liposomal formulation, represented by DLI. All formulations were dosed four times at 1 mg/kg of DOX, with an accompanying amount of 5FUR−W as determined by ratio. The cumulative dosages are far below the reported doses of PEGylated doxorubicin liposomes (25 mg/kg) [50] and free 5FU (200 mg/kg) used preclinically in BALB/c mice [51].

DAFODIL_R=10_ demonstrated remarkable tumor control, but the incorporation of a high amount of 5FUR−W proved toxic. As shown in the pharmacokinetic studies, 5FUR−W has a similar circulation half-life to doxorubicin when administered in DAFODIL. A high ratio, an aggressive dosing regimen, and protection from enzymatic degradation likely led to the toxic buildup of 5FUR−W. Two formulations with lower 5FUR−W:DOX ratios, DAFODIL_R=6_ and DAFODIL_R=2.5_, were tested against DLI. DAFODIL_R=2.5_ had similar efficacy to DAFODIL_R=6_. Additionally, no toxicity was shown in mice treated with DAFODIL_R=2.5_. This indicates that there is likely a window of ratios that are both effective and nontoxic. In our preliminary study, the DAFODIL_R=2.5_ formulation was able to observe a 62.6% reduction in tumor size compared to the untreated control group, and a 33% reduction when compared to the DLI-treated group.

## 5. Conclusions

An improvement in 5-fluorouridine prodrug design led to increased stability and widened the range of drug loading ratios. We were able to identify that ratios greater than 6:1 5FUR−W: DOX in liposomes led to weight loss in mice, while a ratio of 2.5:1 5FUR−W: DOX had increased therapeutic efficacy in the 4T1 murine breast cancer model while maintaining mice body weight. Subsequent evaluation of efficacy and survival rates in more clinically relevant animal models, such as patient derived-xenograft models, would greatly inform on the clinical translatability of DAFODIL. Further safety evaluations are also recommended, including cardiac toxicity and tissue H&E staining, as well as closer analysis of liposome morphology.

## Figures and Tables

**Table 1:** Liposomal size and zeta potential of DAFODIL and DOX liposomes (DOX-L)

Liposome	Size (nm)	PDI	Zeta Potential (mV)
DAFODIL_R=6_	74.7 ± 2.2	0.09 ± 0.01	−25.7 ± 1.4
DAFODIL_R=2.5_	79.9 ± 3.5	0.07 ± 0.05	−24.6 ± 1.2
DOX-L	75.5 ± 2.8	0.05 ± 0.01	−23.3 ± 1.2

**Table 2:** Pharmacokinetic parameters of DAFODIL and DLI.

Formulation	Drug	C_max_ (mmol/L) ^a^	AUC_0__→__t_ (mmol/L*h) ^b^	t_1/2_ (h) ^c^	V_d_ (mL) ^d^	CL (mL/h) ^e^
DAFODIL	5FURW	0.11 ± 0.008	1.15 ± 0.15	8.81 ± 1.43	0.76 ± 0.02	0.06 ± 0.01
	DOX	0.05 ± 0.001	0.62 ± 0.09	10.47 ± 2.02	0.78 ± 0.03	0.05 ± 0.01
DLI	DOX	0.07 ± 0.003	0.78 ± 0.05	14.79 ± 4.11	0.95 ± 0.23	0.04 ± 0.001

^a^ C_max_, plasma concentration maximum; ^b^ AUC, area under the curve representing total drug exposure from t = 0 hr to t = 24 hr; ^c^ t_1/2_, half-life; ^d^ V_d_, volume of distribution; ^e^ CL, total body clearance.

## Data Availability

The data presented in this study are available on request from the corresponding author.

## Supplementary Materials

The following are available online at https://www.mdpi.com/1999-4923/13/1/107/s1, Figure S1. Example HPLC chromatogram of free drugs. (A) 5FUR−W3. (B) Doxorubicin; Figure S2. In vitro cellular toxicity of DAFODIL on both (A) 4T1 cells (500 cells/well) and (B) SKOV3 cells (5000 cells/well); Figure S2. DAFODIL biodistribution is similar to commercial generic PEGylated liposomal doxorubicin, as well as DOX-L; Figure S3. DAFODIL_R=2.5_ tumor response in comparison to DLI extended to 27 days; Table S1. Liposomal dosages in 4T1 animal studies; Table S2. In vitro dose-response parameters of free drug combinations on 4T1 cells. All ratios given are molar ratios, Table S3. In vitro dose-response parameters of liposomes on 4T1 and SKOV3 cells.
