# Supplementary material for: Optimized 5-Fluorouridine Prodrug for Co-Loading with Doxorubicin in Clinically Relevant Liposomes
Source: Pharmaceutics. 2021 Jan 15;13(1):107. doi: 10.3390/pharmaceutics13010107 (PMC7830726; doi:10.3390/pharmaceutics13010107)
Supplement: Supplementary file 1 [file pharmaceutics-13-00107-s001.pdf]

pharmaceutics

Supplementary Materials: Optimized 5-Fluorouridine Prodrug for Co-Loading with Doxorubicin in Clinically Relevant Liposomes

Debra Wu, Douglas Vogus, Vinu Krishnan, Marta Broto, Anusha Pusuluri, Zongmin Zhao, Neha Kapate and Samir Mitragotri

Table S1. Liposomal dosages in 4T1 animal studies.

	5FURW (mg/kg)	DOX (mg/kg)
$DAFODIL_{R=2.5}$	2.4	1
$DAFODIL_{R=6}$	5.8	1
$DAFODIL_{R=10}$	9.7	1

Table S2. In vitro dose-response parameters of free drug combinations on 4T1 cells. All ratios given are molar ratios.

4T1, 500 cells/well		
**Drug/Drug combination**	**IC_{50} (μM)**	**Hill Coefficient**
DOX	$0.004 \pm 6.7\times10^{-4}$	0.41 ± 0.025
5FUR-W	0.061 ± 0.007	0.95 ± 0.092
1:5 5FUR-W:DOX	0.016 ± 0.002	0.48 ± 0.029
1:1 5FUR-W:DOX	$0.011 \pm 7.9\times10^{-4}$	0.51 ± 0.019
5:1 5FUR-W:DOX	0.040 ± 0.004	0.038 ± 0.004
$5FUR-W_3$	0.040 ± 0.006	0.69 ± 0.067
1:5 $5FUR-W_3$:DOX	0.033 ± 0.003	0.72 ± 0.051
1:1 $5FUR-W_3$:DOX	$0.004 \pm 5.0\times10^{-4}$	0.43 ± 0.020
5:1 $5FUR-W_3$:DOX	0.038 ± 0.004	0.59 ± 0.033

Table S3. In vitro dose-response parameters of liposomes on 4T1 and SKOV3 cells.

4T1, 500 cells/well		
**Formulation**	**IC_{50} (μM)**	**Hill Coefficient**
DOX-L	1.61 ± 0.24	0.67 ± 0.07
5FURW-L	0.094 ± 0.02	0.65 ± 0.08
R=2.5 5FURW-L:DOX-L	0.030 ± 0.003	0.60 ± 0.04
$DAFODIL_{R=2.5}$	0.06 ± 0.007	0.74 ± 0.06
**SKOV3, 5000 cells/well**		
**Formulation**	**IC_{50} (μM)**	**Hill Coefficient**
DOX-L	55.16 ± 9.57	0.46 ± 0.05
5FURW-L	22.02 ± 3.90	0.43 ± 0.04
R=2.5 5FURW-L:DOX-L	24.37 ± 5.95	0.42 ± 0.05
$DAFODIL_{R=2.5}$	8.76 ± 1.47	0.56 ± 0.05

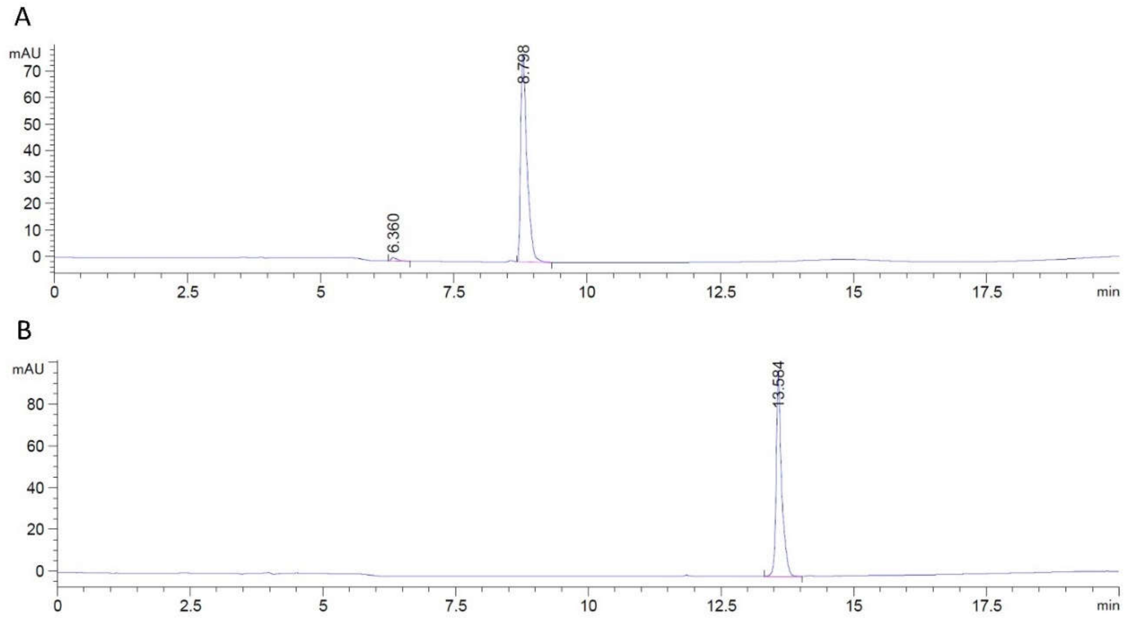

Figure S1. Example HPLC chromatogram of free drugs. **(A)** 5FUR-W3. **(B)** Doxorubicin.

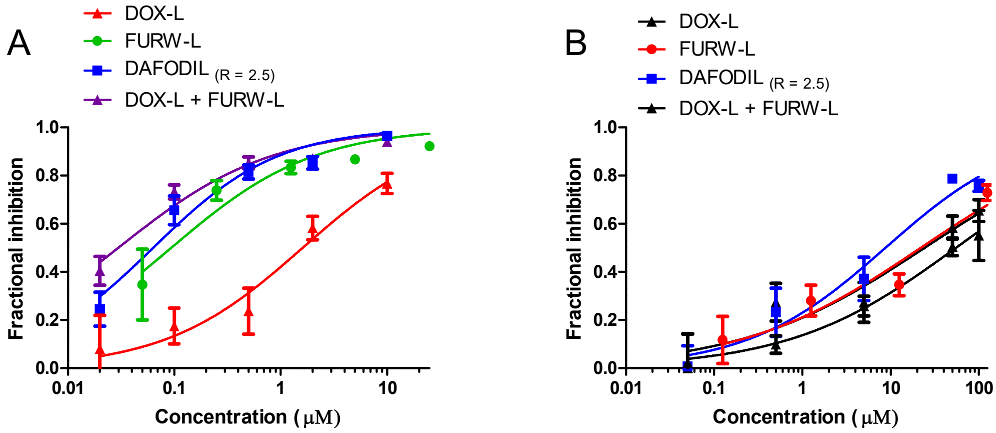

Figure S2. In vitro cellular toxicity of DAFODIL on both **(A)** 4T1 cells (500 cells/well) and **(B)** SKOV3 cells (5000 cells/well).

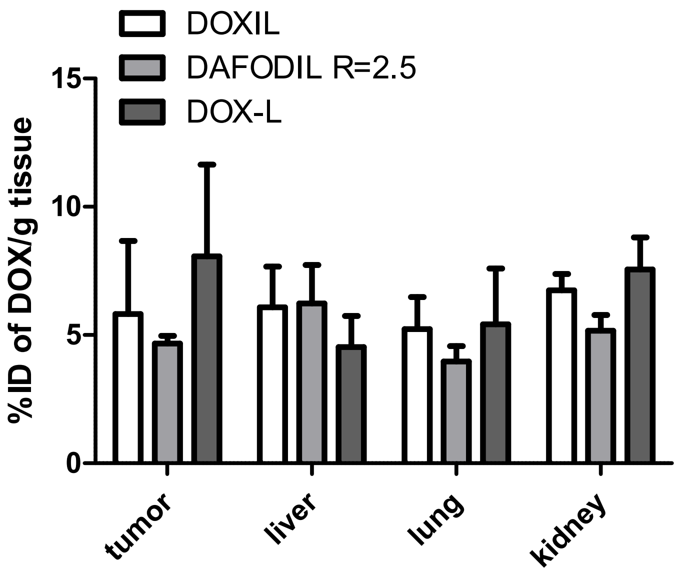

Figure S3. DAFODIL biodistribution is simmilar to commercial generic PEGylated liposomal doxorubicin, as well as DOX-L.

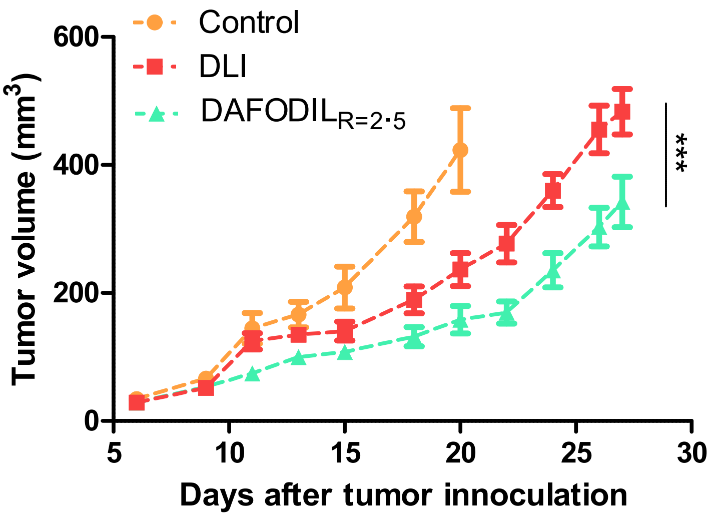

Figure S4. DAFODIL R=2.5 tumor response in comparison to DLI extended to 27 days.
